# A Folding Pathway-Dependent Score to Recognize Membrane Proteins

**DOI:** 10.1371/journal.pone.0016778

**Published:** 2011-03-01

**Authors:** Hamid Hadi-Alijanvand, Maryam Rouhani, Elizabeth A. Proctor, Nikolay V. Dokholyan, Ali A. Moosavi-Movahedi

**Affiliations:** 1 Institute of Biochemistry and Biophysics, University of Tehran, Tehran, Iran; 2 Genetics Medicine, Department of Biochemistry and Biophysics, University of North Carolina at Chapel Hill, Chapel Hill, North Carolina, United States of America; University of South Florida College of Medicine, United States of America

## Abstract

While various approaches exist to study protein localization, it is still a challenge to predict where proteins localize. Here, we consider a mechanistic viewpoint for membrane localization. Taking into account the steps for the folding pathway of α-helical membrane proteins and relating biophysical parameters to each of these steps, we create a score capable of predicting the propensity for membrane localization and call it FP_3_mem. This score is driven from the principal component analysis (PCA) of the biophysical parameters related to membrane localization. FP_3_mem allows us to rationalize the colocalization of a number of channel proteins with the Cav1.2 channel by their fewer propensities for membrane localization.

## Introduction

Calcium influx plays a significant role in controlling a variety of cellular functions, and is mainly carried out by voltage-gated Ca^2+^ channels [1], [2]. Voltage-gated L-type Ca^2+^ channels (LTCCs) are involved in the regulation of muscle contraction, hormone secretion, neural excitability, gene expression and neurotransmitter release. LTCC channels consist of four isoforms: Ca_v_1.1, Ca_v_1.2, Ca_v_1.3 and Ca_v_1.4, of which Ca_v_1.2 and Ca_v_1.3 are more distributed and localize in diverse tissues [3]–[11]. Ca_v_1.2 makes up at least 75–80% of the LTCCs of the brain [12]–[15]. Many types of channels and receptors correlate functionally and spatially with Ca_v_ channels.

Small conductance Ca^2+^- activated K^+^ channels (SK channels) are a group of channels affected by Ca^2+^ influx and involved in afterhyperpolarizations (AHPs) following the membrane action potentials.[16]–[22]. Kohler et al. cloned these channels in 1996 and found three subtypes: K_Ca_2.1 (SK1), K_Ca_2.2 (SK2) and K_Ca_2.3 (SK3) [22]. These channels are voltage-independent but highly sensitive to [Ca^2+^]_i_ due to the C- terminal bound calmodulin protein [16], [23]–[28]. The channels are mainly located in the central and peripheral nervous systems [29]–[33].

Interestingly, the SK channels are specifically coupled to and activated by the Ca^2+^ channels, including LTCCs [16], [34]. During the depolarization periods, the LTCC channels mediate the Ca^2+^ influx. The subsequent binding of calcium to calmodulin leads to the conformational change and opening of the SK channels that causes the efflux of potassium ions. Thus, a close physical and functional relationship exists between these two types of channels. Lu et al. were the first who indicated the coupling of LTCC and SK channels via cytoskeleton proteins [35]. They demonstrated that the SK2 and Ca_v_1.2 or Ca_v_1.3 channels are linked via an important component of the actin cytoskeleton, α-actinin2.

Another group of ion channels co-localized with LTCCs are the glutamate receptors, located in postsynaptic sites of excitatory synapses. N-methyl-D aspartate receptors (NMDARs) and α-amino-3-hydroxy-5-methyl-4-isoxazole propionate receptors (AMPARs) are members of the glutamate receptor channel superfamily, located in close proximity to the Ca_v_1.2 channels [36]–[38]. Reports indicate the involvement of both LTCCs and glutamate receptors in the constitutive increase in synaptic transition [39]–[42].

Existing methods for the scaling of membrane localization propensity use algorithms such as hidden Markov models (HMMs) and supported vector machines (SVMs) to recognize protein sequences that have the potential to sub-localize within the membrane [43]. However, these methods do not consider the causal folding pathway involved in recognition. We introduce the **F**olding **P**athway-based **P**rotein **P**ropensity for membrane (FP_3_mem) score that is tightly associated with the tendency of proteins for being α-helical plasma membrane proteins. We use this score for interpreting the colocalization of the Ca_v_1.2 channel with the rat SK (rSK) channels, and with the AMPAR and NMDAR receptors in Eubacteria and Archea taxa and in vertebrate classes including Fishes, Amphibia, Aves and Mammalia. Our data characterized the Ca_v_1.2 as having a high propensity for localization within the plasma membrane together with other willing channels, which supports the hypothesis that the Ca_v_1.2 is an anchor for the membrane proteins in its close proximity.

## Methods

The sequences of rSK1 (gi 9506831), rSK2 (gi 9506833), rSK3 (gi 31543039), Ca_v_1.2 (gi 158186633), α-actinin (gi 1142640), AMDAR (gi 167001419) and NMDAR (gi 11038637) were taken from the NCBI protein database in FASTA format. Subsequently, using the NCBI protein BLAST service and the Blosum62 matrix [44], we found sequences homologous to the abovementioned proteins from the protein non-redundant database in the Archea (taxid 2157) and Eubacteria taxa (taxid 2), as well as vertebrate classes including Fishes (taxid 7898), Amphibia (taxid 8292), Aves (taxid 8782) and Mammalia (taxid 40674) (Table S1).

We calculate the thermodynamic, biophysical, and structural parameters ΔCp (change in specific heat), ΔCp(hyd) (change in hydration specific heat), ΔG(hyd) (change in Gibbs energy of hydration), ΔG(oct) (change in free energy of transfer from water to octanol), ΔG(wif) (change in free energy of transfer from water to POPC interface), ΔΔG(α-helix), GG4Br, ΔH(hyd) (change in enthalpy of hydration) and kProt for the sequences obtained from the BLAST. We consider ΔCp, ΔG(hyd) and ΔH(hyd) as parameters characterizing protein properties in the water phase. ΔG(oct), ΔG(wif) and ΔΔG(α-helix) have a role in the transition of proteins from the aqueous phase to the lipid phase. Finally, ΔCp(hyd), GG4Br, and kProt explain the behavior of proteins in the lipid phase.

We perform this calculation using the Hamid, Ali akbar, Maryam Data Analyser Machine (HAMDAM) software (freely available upon request). We calculate the hydration (hyd) parameters ΔCp(hyd), ΔG(hyd) and ΔH(hyd) of each sequence using the following equations [45]–[47]:
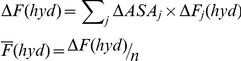
Where ΔX refers to the change in X from the native state to the unfolded state, ΔF(hyd) represents each of the three parameters, j is the residue position, ASA stands for the accessible surface area, and *n* represents the total number of residues in each sequence.

We obtain ΔCp from the following equation [48]:
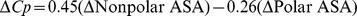
In order to calculate the ΔG(oct) [49], ΔG(wif) [50], [51], ΔΔG(α-helix) [52], GG4Br [53], and kProt [54] (all indicated with a “W” after the parameter name in figures), we employ the Sliding Window Recognizer (SWR) procedure [55]. This procedure reads the protein sequence within a window of a given number of residues and computes the parameters for the amino acids within that window, then slides forward one residue and repeats the process. We choose a window of 10 residues and calculate the parameter average for each window. Then we report the average of averages over all windows. In the case of the ΔΔG(α-helix) parameter, although proline residues are considered helix breakers, their behavior differs in membrane proteins [56], which led us to consider this amino acid as a helix maker within this subset of proteins. For calculation of the GG4Br parameter, the number of GXXXG[I/V] motifs are counted in each window. We perform Anova and PCA analysis using the free software PSPP (http://www.gnu.org/software/pspp).

To produce alkaline phosphatase (APHO)18A3L, APHO16A5L and APHO14A7L sequences, three peptide constructs generate with the 18A3L, 16A5L and 14A7L amino acids compositions. In order to consider different sequences for each of the three amino acid compositions, we generate 2000 random sequences for each peptide and insert them to the corresponding site in alkaline phosphatase.

## Results and Discussion

Other studies have previously pointed out the association of Ca_v_ with SK channels in membranes [16], [34], [35]. Lu et al. demonstrated the connection of these two channels via the α-actinin protein [35]. On the other hand, the ion conductance through the membrane and the localization within the membrane of the SK channel was disrupted in Ca_v_ null mutant mouse. The authors suggested that the Ca_v_ channel could act as an anchor for the SK channel at the membrane. For integral membrane proteins containing transmembrane region(s), an essential requirement for functionality is localization within the membrane. Our goal is to quantify the tendency of Ca_v_ and SK channels for localizing within the membrane.

**Figure 1:**
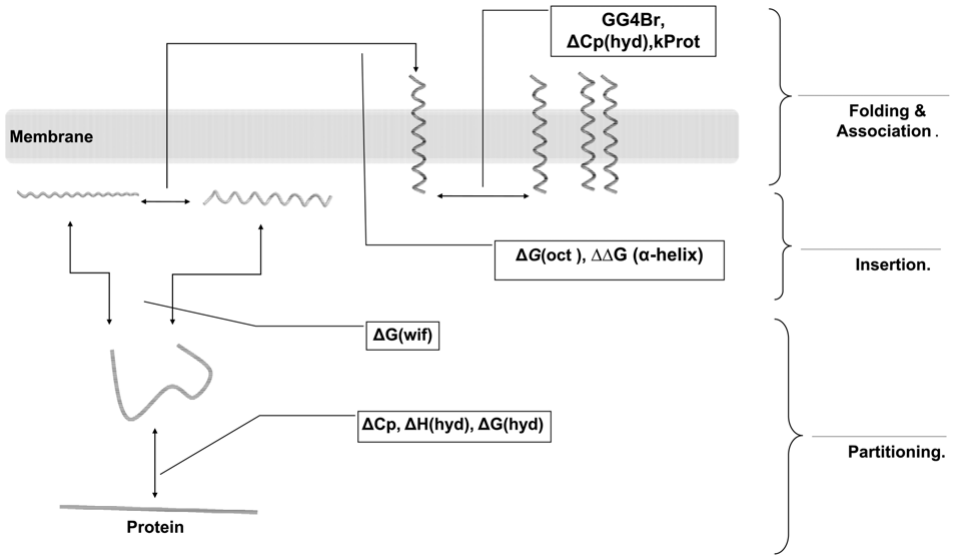
Alpha helical membrane protein folding pathway. The four step-folding pathway of membrane proteins declares the critical factors that play a role in the folding of α-helical membrane proteins. Partitioning includes parameters involved in protein partitioning in the lipid-water interface. The insertion stage contains parameters required for a peptide to insert into plasma membrane. The final folding and association stages indicate critical parameters for the membrane protein folding and packing.

White and Wimley mentioned that the folding process of membrane proteins could be divided into four steps, including partitioning, insertion, folding, and association [49]. We consider several biophysical parameters for each of these steps. Two parameters (ΔG(oct) and ΔG(wif)) were previously provided for two of these steps [49], [51]. We consider other parameters related to each step in our computation (Figure 1). As a control, we compute these parameters for the non-membrane protein alkaline phosphatase (APHO). We also calculate parameters for three varieties of alkaline phosphatase: APHO18A3L, APHO16A5L and APHO14A7L. These alkaline phosphatases localize in the E-coli inner membrane with the aid of three inserted peptides. Each peptide is composed of only two types of amino acids, Alanine (A) and Leucine (L). The experimentally determined tendency of alkaline phosphatase for localizing in the membrane is in direct correlation with the number of leucine residues in the inserted peptides [57], [58].

### The partitioning step

**Figure 2:**
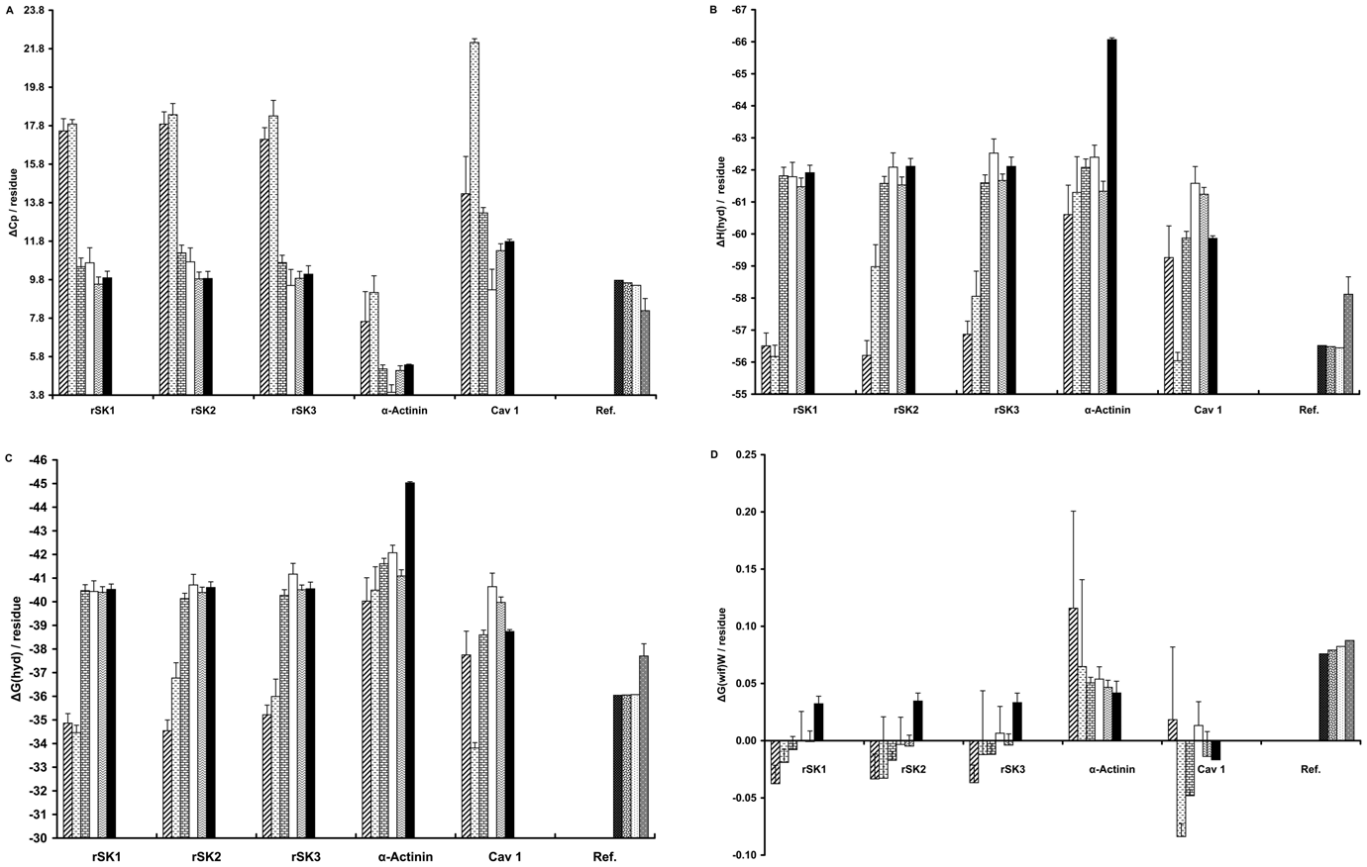
The partitioning step parameters. The changes in parameters that describe the partitioning stage are indicated for bacteria and vertebrate proteins, A) the specific heat capacity, a measure of protein stability, B, C) the specific enthalpy and Gibbs energy of hydration, respectively. D) Free energy change of transfer from water to the POPC interface. Ref. stands for reference proteins. Error bars indicate the SEM for the parameters of each protein. The bar patterns represent Archea: wide upward diagonal, Eubacteria: dashed horizontal, Fishes: horizontal brick, Amphibia: white, Aves: wave, Mammalia: black, APHO 14A7L: black dotted white, APHO 16A5L: white grained black, APHO 18A3L: white dotted black, and APHO: gray dotted white.

The “partitioning” step, the partitioning of proteins between lipid and water phases in the lipid-water interface, can be described by the ΔCp, ΔH(hyd), ΔG(hyd), and ΔG(wif) parameters. A membrane protein should not have a stable fold before insertion into the membrane. This property is specified by the protein heat capacity ΔCp. A more positive ΔCp indicates lower stability, and thus a lower propensity to be in the folded state in the water phase [55]. The ΔCp of the alkaline phosphatases (the reference proteins, “Ref”) that contain inserted peptide is more positive than the ΔCp of the alkaline phosphatase (Figure 2A), representing a difference between the primitive forms of life (Archea and Eubacteria) and vertebrates. In vertebrates, the ΔCp is lower and thus the propensity for folding in the water phase is higher than in bacteria. Therefore, for prokaryotes, the partitioning parameter is more favorable for membrane localization when compared to vertebrates. Predictably, the α-actinin homologous proteins have fewer propensities for unfolding in water with respect to channels.

Another parameter involved in the partitioning step is the hydration enthalpy change ΔH(hyd). This parameter is a scale of the hydrophilic interaction of the unfolded state. A more negative ΔH(hyd) indicates a higher tendency of the protein to be in the unfolded state in water [45], [46]. In the case of the reference proteins, because the three types of peptides are composed solely of leucine and alanine residues, the amount of hydrophilic interactions is reduced (Figure 2B). The α-actinin homologous proteins have a dramatically higher tendency for unfolding in Mammalia than other organisms. For the rSK channel homologous proteins, the tendency of unfolding in water is higher in vertebrates than in bacteria. This tendency indicates that according to this partitioning parameter, in comparison to the prokaryotic protein, the vertebrate protein is far from folded state in water. This favors folding of the vertebrate protein in non-aqueous environment. It may be presumed that there is a discrepancy between the ΔCp- and ΔH(hyd)-derived partitioning parameters in each group of proteins(Panels 2-A and 2-B). However, as the ΔCp is generally determined by nonpolar residues and the ΔH(hyd) parameter by polar residues, the difference between the ratio of polar to nonpolar residues is the root of this apparent dissimilarity. Each of these parameters is weighted later.

An additional parameter affecting the partitioning step can be the hydration free energy change ΔG(hyd). A membrane protein must dehydrate before entering the plasma membrane. Since the ΔG(hyd) is a scale of the propensity for hydration, the more positive this parameter, the easier is the dehydration process [45], [59]. The propensity of rSK channel homologous proteins for dehydration is less in vertebrates than in bacteria (Figure 2C), indicating that the partitioning affected by this parameter happens more difficult in vertebrate classes than in Archea and Eubacteria. Among all studied proteins, mammalian α-actinin homologous proteins have the least propensity for dehydration (Figure 2C).

The last parameter that we incorporate, influencing the partitioning of proteins between the water and lipid phases is the ΔG(wif), was introduced by White et al. [51]. This parameter represents the free energy change for the transfer of the protein in the unfolded state from the bulk water to the lipid-water interface. Nonpolar interactions with water and electrostatic interactions with the lipid head groups are the important interactions taking part in this process [49], [60]. The more negative the ΔG(wif), the higher is the affinity of the unfolded state to enter the interface [61]. This parameter is small for the rSK homologous proteins in the taxa and classes other than Mammalia (Figure 2D). Thus, the propensity for entering the lipid-water interface is low in mammalian rSK channels but not in the mammalian Ca_v_ channels. However, when we consider all four parameters involved in the partitioning step, we cannot simply conclude which of the two types of channels is more efficient in this process.

### The insertion step

**Figure 3:**
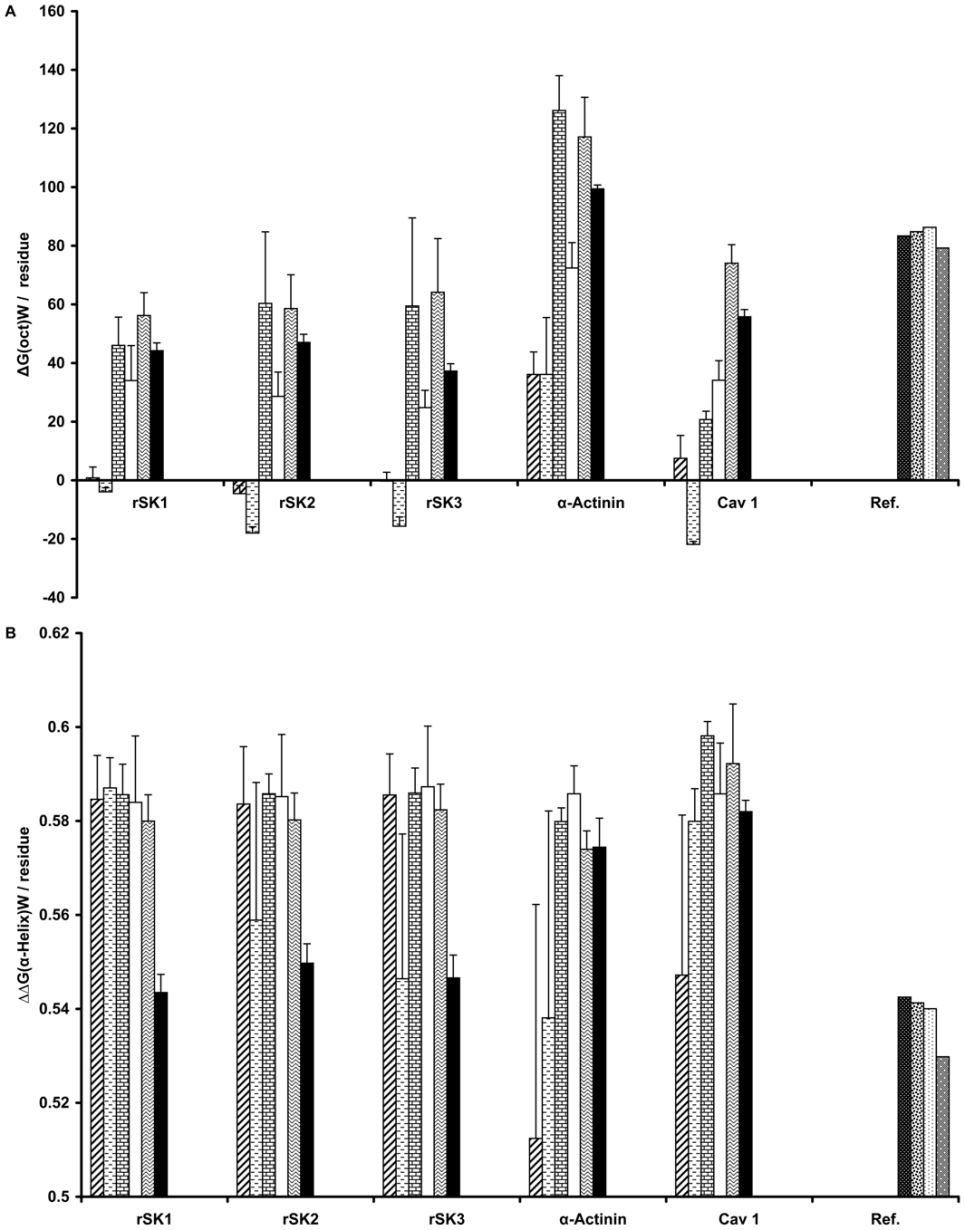
The insertion step parameters. The changes in parameters describing the insertion stage are shown for bacterial and vertebrate proteins. The vertical axes are the mean value of the corresponding parameter average for the window that slides along the protein sequence. A) The variation of ΔG(oct) is illustrated, which is a whole residue hydrophobicity scale and a sign of the protein membrane propensity. B) The alpha helix propensity is a measure of the tendency to form alpha helix, which is characterized by ΔΔG(α-helix). Ref. stands for the reference proteins. The error bars indicate the SEM for the parameters of each protein. The bar patterns represent Archea: wide upward diagonal, Eubacteria: dashed horizontal, Fishes: horizontal brick, Amphibia: white, Aves: wave, Mammalia: black, APHO 14A7L: black dotted white, APHO 16A5L: white grained black, APHO 18A3L: white dotted black, and APHO: gray dotted white.

The second step in the folding of membrane proteins is the “insertion” of the protein into the membrane. In this step, a protein enters the lipid phase from the lipid-water interface. Two parameters are associated with this step: the ΔG(oct) and the ΔΔG(α-helix) [49], [52], [62]. In their study, the White group utilized octanol-saturated water as the lipid-like phase and introduced the ΔG(oct) parameter. More negative values of the ΔG(oct) correlate with a higher tendency of the protein for transferring to the lipid phase. For channels, this insertion parameter is more favorable in prokaryotes than in vertebrates (Figure 3A). We infer that the transition from the interface to the lipid phase acts as an obstacle for vertebrate channels in the process of membrane localization.

In the interface, where the important step of insertion into the membrane takes place, formation of disordered structures is more probable than formation of helical structures [60], [63]. Therefore, protein structures are more likely to become α-helical after insertion into the membrane because of the low membrane dielectric constant [49], [64], [65]. Because the hydrophobic core of the membrane has a high affinity for exposed hydrophobic groups of proteins [66], if an α-helix is stable in the water phase it would not form hydrophobic interactions with the membrane core. The ΔΔG(α-helix) specifies the propensity for the formation of a stable α-helix structure in the water phase. More positive values of this parameter correlate with a lower propensity for α-helix formation in water, and thus are more favorable for the insertion of the protein into the membrane. For rSK channel homologous proteins, this parameter decreases in Mammalia (Figure 3B) and disturbs the insertion step.

These parameters do not have the same effects in the localization of each evolutionary class of protein within the plasma membrane (Figures 2 and 3). While some parameters support the membrane localization of the homologous sequences of one protein, others impede this process. Therefore, all parameters should be weighted accordingly when calculating the membrane localization score.

### Folding and association steps

**Figure 4:**
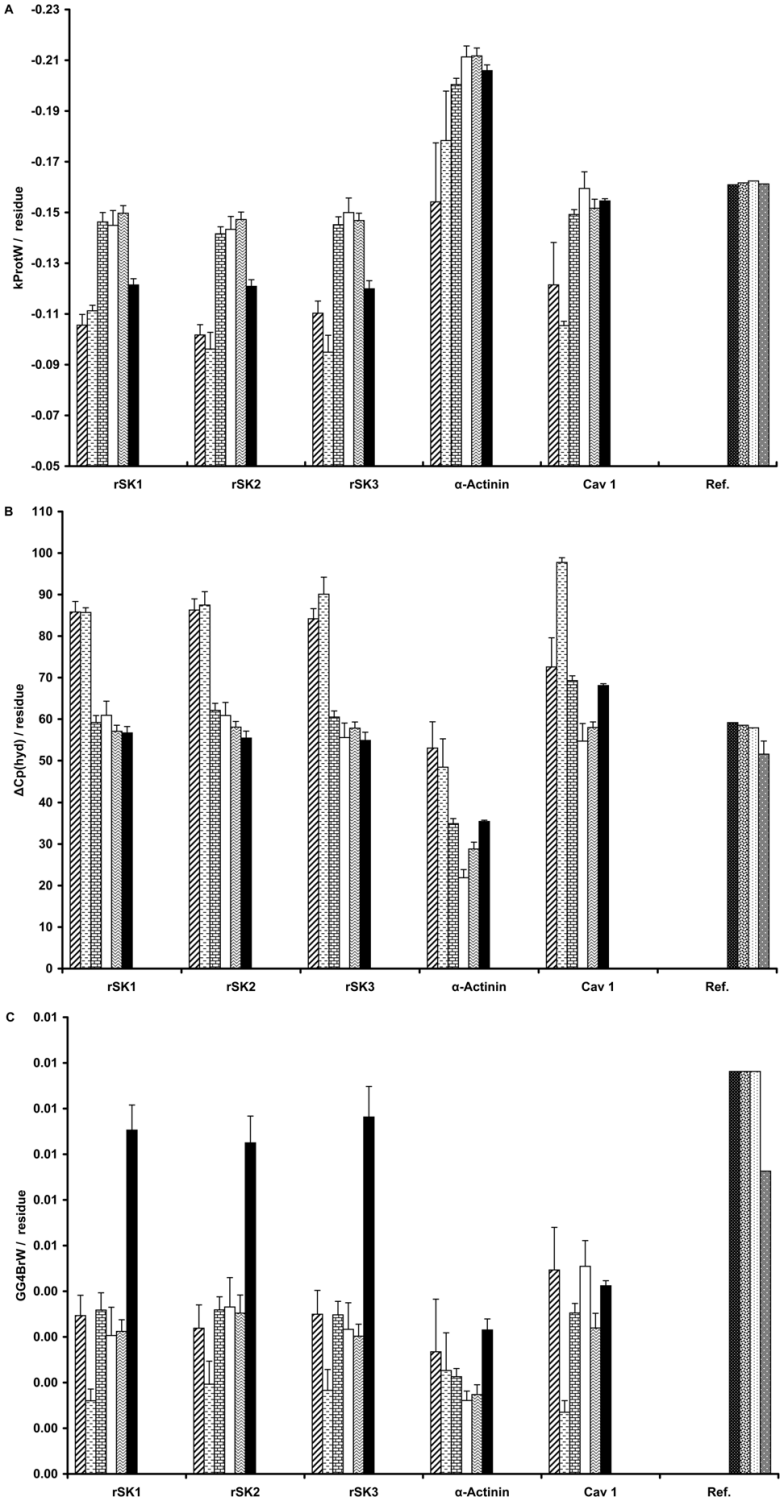
The folding and association stages parameters. The changes in parameters describing the folding and association stages are shown for bacteria and vertebrates proteins. In the A and C panels, the vertical axes are the mean value of the corresponding parameter average for the window that slides along the protein sequence. A) The kProt changes are shown in this panel. kProt is a factor to elucidate α-helix membrane protein topology. B) The specific heat capacity of hydration for the channels, references and actinin is shown in this board. ΔCp(hyd) points out the hydrophobic patches. C) The occurrence number of the GG4Br motif in windows is defined as a scale for helix packing. Ref. stands for the reference proteins. The error bars indicate the SEM for the parameters of each protein. The bar patterns represent Archea: wide upward diagonal, Eubacteria: dashed horizontal, Fishes: horizontal brick, Amphibia: white, Aves: wave, Mammalia: black, APHO 14A7L: black dotted white, APHO 16A5L: white grained black, APHO 18A3L: white dotted black, and APHO: gray dotted white.

Based on the four-step model, a protein obtains its final folded state in the membrane and, if necessary, gains its final function by association with other subunits. An important feature of alpha helical membrane protein folding is protein topology, which can be either single-span or multi-span, represented by the parameter kProt [54]. More negative kProt values correlate with higher protein tendency for becoming multi-span. Prokaryotes show lower tendency than vertebrates for multi-span topology (Figure 4A). In the case of rSK channel homologous proteins, the tendency for becoming multi-span is less in mammals than in other vertebrates. The definition of kProt parameter is founded on the properties of membrane proteins. Therefore, this parameter does not provide information about the topology of non-membrane proteins including alkaline phosphatase and α-actinin homologous proteins (Figure 4A).

Due to the low dielectric constant of the membrane, hydrogen bond rich structures such as α-helices are more probable in membrane proteins. In order to attain more stability and generate a specific function, the α-helices pack together in a manner such that a stable helix can stabilize an adjacent unstable helix [65], [67], [68]. The packing of α-helices is caused by two factors: superficial hydrophobic patches working as glue, and spatial fitting of the α-helices similar to lock and key model. The ΔCp(hyd) parameter represents superficial hydrophobic patches. A more positive ΔCp(hyd) indicates a more exposed hydrophobic patch [45]. The combined surface area of superficial hydrophobic patches, which is a scale for the association of membrane α-helices, is high for the rSK channel homologous proteins in comparison to the α-actinin homologous proteins (Figure 4B). Furthermore, the surface area of superficial hydrophobic patches, and therefore the tendency for association indicated by this factor, decreases from prokaryote to vertebrate organisms, especially in the rSK channel homologous proteins (Figure 4B). The second packing factor, the spatial fitting of membrane α-helices, can be quantified by measuring the frequency of the GXXXG[I/V] motif in the proteins using the GG4Br parameter [53], [69]. For rSK channel homologous proteins, the frequency of the GXXXG[I/V] motif is much higher in Mammalia as compared to other classes (Figure 4C). We conclude that in the mammalian rSK channels, the spatial fitting of α-helices plays a more significant role in packing than the superficial hydrophobic patches.

### The FP_3_mem score

Not all parameters involved in protein localization within the plasma membrane change in the same functional direction over evolution (Figures 2, 3 and 4). Hence, we create a parameter that in addition to including all significant parameters previously discussed, is able to determine the tendency of localization of proteins within the membrane. This parameter can also be used as a scale for the comparison of membrane localization between proteins of interest. In order to fulfill this purpose, all parameters that participate in membrane localization should be weighted according to their contributions. We use principal component analysis (PCA) to obtain proper weights for each parameter [70]. We consider four principal components (PC) and utilize the proposed correlation coefficients for each parameter in each PC for constructing a factor representing the tendency of proteins for membrane localization. We name this factor the **F**olding **P**athway-based **P**rotein **P**ropensity for membrane (FP_3_mem). The HAMDAM software calculates the FP_3_mem based on the following formula:
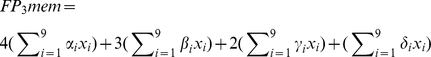
In this equation, the set of x*_i_* represents the nine parameters (*i* = 1 to 9) for the rSK and Ca_v_1.2 channel homologous proteins. Here, α, β, γ, and δ correspond to the correlation coefficients of each parameter in PC1, PC2, PC3 and PC4 respectively. More details are supplied in Table S2.

**Figure 5:**
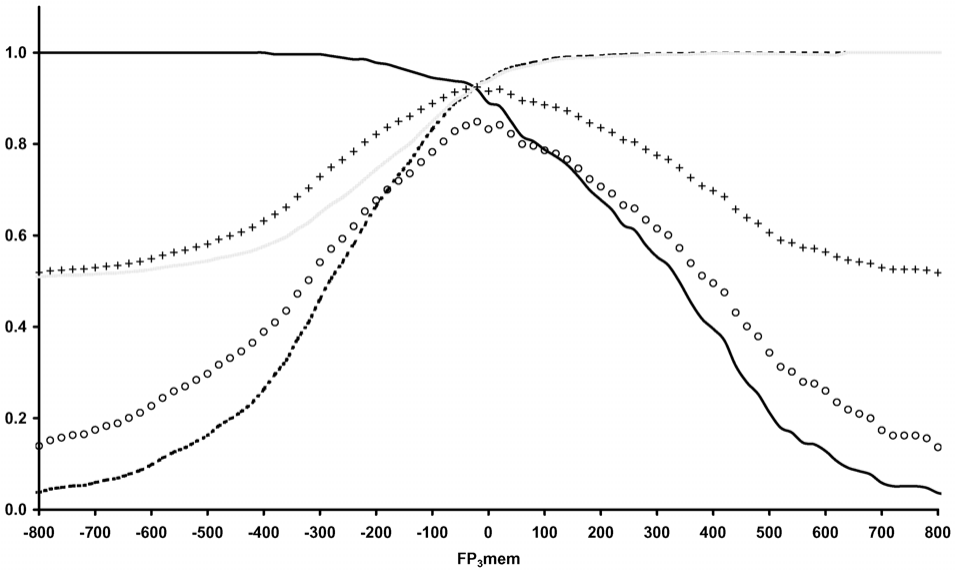
The dependence of statistical descriptors to the FP_3_mem cut-offs. Variations of common statistical descriptors (which are used to evaluate a new predictor) in response to the FP_3_mem cut-off changes are depicted. Continues dark line, discontinues line, continues gray line, plus and circle symbol stand for sensitivity, specificity, PPV, accuracy and MCC parameters respectively.

We calculate the FP_3_mem for all sequences in the TMA dataset [71], which contains 273 transmembrane α-helix-containing sequences from PDB structures, as well as for the sequences of a set of human soluble proteins, documented as cell fraction, obtained from Uniprot. These sets serve as references for membrane proteins and non-membrane proteins, respectively. In order to define a cut-off for FP_3_mem values that discriminates membrane proteins from non-membrane proteins, we evaluate the following parameters [72],[73] over a wide range of FP_3_mem cut-offs (Figure 5):

TP = the fraction of membrane proteins recognized as membrane proteins.
FN = the fraction of membrane proteins falsely recognized as non-membrane proteins.
TN = the fraction of non-membrane proteins recognized as non-membrane proteins.
FP = the fraction of non-membrane proteins falsely recognized as membrane proteins.
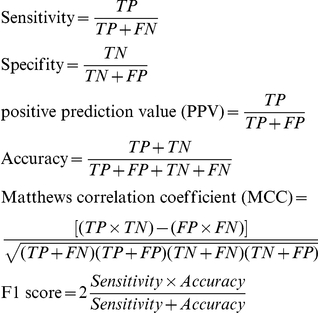

Based on these results and the corresponding ROC curve (Figure S1), we set our cut-off at FP_3_mem = −31, and consider the proteins with FP_3_mems values higher than −31 as membrane proteins.

In order to evaluate the FP_3_mem capability for discriminating membrane proteins, we calculate the abovementioned statistical parameters for several datasets (Table S3). One of these datasets, the Moller dataset, includes three levels of trust to SWISSPROT transmembrane annotation: A, B, and C [74] (Table 1). The accuracy of FP_3_mem is best for the B dataset, in which membrane localization of dataset members has been proven by experimental evidence. If we omit mitochondrial membrane proteins from the A dataset, in which protein structures have been determined by x-ray crystallography, the accuracy for the A dataset increases (Table 1). We conclude that in mitochondria, the process of membrane localization may not be similar to the four-step membrane protein folding process (Figure 1).

**Table 1:** FP_3_mem-based statistical values for different databases.

		Moller				MPtopo				
	TMA	A	B	C	A^m^	MPT	MPT1D	MPT3D	MPT3D^m^	Alpha
**F1**	0.9	0.8	0.9	0.7	0.9	0.9	0.9	0.9	0.9	0.9
**Specificity**	0.9	0.9	0.9	0.9	0.9	0.9	0.9	0.9	0.9	0.9
**PPV**	0.9	0.9	0.9	0.9	0.9	0.9	0.9	0.9	0.9	0.9
**Accuracy**	0.9	0.8	0.9	0.8	0.9	0.9	0.9	0.9	0.9	0.9
**MCC**	0.8	0.7	0.8	0.6	0.8	0.8	0.8	0.8	0.8	0.8

To evaluate the FP_3_mem efficiency, we calculated the statistical parameters in various databases. These datasets contain information about transmembrane proteins obtained from x-ray crystallography or other experimental methods used to verify the 3D structure. MPT is the whole MPtopo database. MPT1D and 3D are sub populations of MPT. The m superscripts indicate that the mitochondrial proteins are omitted from the dataset.

We take another dataset from the MPtopo database (Table 1). Based on whether the protein helix bundles are determined from three dimensional structure or by biochemical experimental methods, Jayasinghe et al. have divided the MPtopo database into 3D and 1D datasets respectively [75]. When we omit mitochondrial membrane proteins from the 3D dataset, the accuracy increases. The lipid context of mitochondrial membrane proteins is different from that of proteins in the plasma membrane [76]. This difference may cause a different pathway of membrane protein folding.

The last dataset that we consider is that of alpha, which is taken from the July 9, 2010 version of the PDBTM [77]. The non-redundant alpha dataset consists of all α-helical transmembrane proteins in the PDB. The calculated statistical factors are also near to one for this dataset, which confirms the accuracy of the FP_3_mem score in distinguishing α-helical transmembrane proteins.

We calculate FP_3_mem for the membrane proteins used in training the PSORTb 3.0 predictor algorithm [78] (Table 2). FP_3_mem has a high efficiency in recognizing prokaryotic membrane proteins. The eSLDB database annotates the eukaryotic proteomes of various organisms based on their cellular localizations [79]. We compute the FP_3_mem score for a group of human, nematode (*Caenorhabditis elegans*), and yeast (*Sacharomyces cerevisiae*) transmembrane proteins that have been experimentally annotated (Table 3). Statistical parameters again confirm the efficiency of FP_3_mem in distinguishing membrane proteins.

**Table 2:** FP_3_mem-based statistical values for a prokaryotic dataset.

	PSORTb 3.0		
	Archea	Bacteria (Gram)	
		+	−
**F1**	0.9	0.7	0.8
**Specificity**	0.9	0.9	0.9
**PPV**	0.9	0.9	0.9
**Accuracy**	0.9	0.8	0.9
**MCC**	0.8	0.6	0.7

To evaluate the FP_3_mem efficiency, we calculated the statistical parameters for the PSORTb3.0 [78] trained sets. PSORTb3.0 is trained on the plasma membrane proteins of prokaryotes selected by searching the SWISSPROT sequence annotations.

**Table 3:** FP_3_mem-based statistical values for a eukaryotic dataset.

	eSLDB-TM		
	Human	Nematode	Yeast
**F1**	0.6	0.6	0.7
**Specificity**	0.9	0.9	0.9
**PPV**	0.8	0.9	0.9
**Accuracy**	0.7	0.7	0.8
**MCC**	0.4	0.5	0.6

To evaluate the FP_3_mem efficiency, we calculated the statistical parameters for the eSLDB [79]. This database contains the whole proteome of many eukaryotes. FP_3_mem identifies the transmembrane proteins of human, yeast and nematode from the database with the indicated efficiencies.

There are several methods for determining the localization of proteins in different regions of the cell, including the plasma membrane. Some commonly used methods include CELLO, which utilizes only the primary structure of proteins [80], pTARGET, which utilizes the amino acid and domain compositions [81], ProteomeAnalyst, which uses the homology of the sequences [82], WoLFPSORT, which makes use of the amino acid composition and the sequence homology [83], and MultiLoc, which employs signal sequences, motifs, and amino acid compositions [84]. Teasdale compared the capacity of these methods for determining the localization of proteins of two datasets, LOC2145 and SP3763 [85]. We calculate the sensitivities and specificities of FP_3_mem and other methods in distinguishing the membrane proteins of these two datasets (Table 4). FP_3_mem has the highest specificities as well as reasonable sensitivities. Binary predictors encounter a common problem of disadvantaged specificities despite good sensitivities [86]. However, FP_3_mem possesses high specificities. Our method, which is based on biophysical parameters of the membrane protein folding pathway, in this regard outperforms the existing methods.

**Table 4:** Comparison of the FP_3_mem efficiency with other methods.

	CELLO		MultiLoc		Proteome Analyst		pTarget		woLFPSORT		FP3mem	
	Sen	Spe	Sen	Spe	Sen	Spe	Sen	Spe	Sen	Spe.	Sen	Spe
**SP3763 ^plasma membrane^**	0.4	0.3	0.4	0.3	0.1	0.4	0.4	0.3	0.5	0.4	0.4	0.8
**LOC2145 ^plasma membrane^**	0.4	0.6	0.5	0.8	0.2	0.8	0.5	0.7	0.5	0.7	0.4	0.8

The FP_3_mem efficiency is compared with other methods on the same datasets that are common for predicting protein membrane localization. Sen and Spe are the abbreviation of sensitivity and specificity respectively.

In order to enter membrane, proteins pass different stages. The superiority of FP_3_mem with respect to other methods comes from the fact that we relate the folding pathway stages of membrane proteins to the representative physical parameters and do predictions with a mechanistical viewpoint which was absent in previous sequence-based methods.

**Figure 6:**
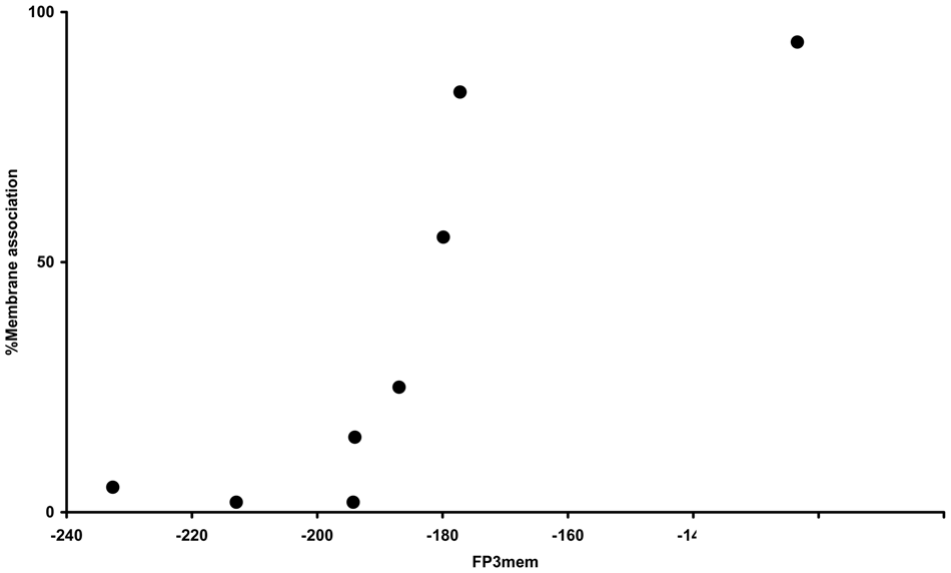
The correlation between FP_3_mem and the amount of membrane associations. A non-linear correlation exists between FP_3_mem and the amount of membrane associations. The FP_3_mem of CN, CNA, CNAA, CNLA1 (APHO 18A3L), CNLA2 (APHO 16A5L), CNLA3 (APHO 14A7L), CNL and CNLL [57] are plotted against the experimentally determined membrane association.

The abovementioned databases and methods provide information about whether a protein localizes within the plasma membrane, but cannot resolve the membrane association (the propensities for membrane localization) of two transmembrane proteins. A probable reason for the co-localization of physically interacting membrane proteins is that a protein with a high membrane association can compensate the low membrane association of its partner. Hence, we examine the capacity of FP_3_mem in quantifying the membrane association of proteins using the alkaline phosphatase variants whose membrane associations were determined empirically [57], [58]. The relationship between the FP_3_mem and membrane association of these variants is direct and non-linear (Figure 6). Therefore, FP_3_mem is not only capable of recognizing α-helical transmembrane proteins with a high efficiency, but also can be a scale for membrane association propensity.

### The membrane proteins co-localized with Ca_v_ channel

**Figure 7:**
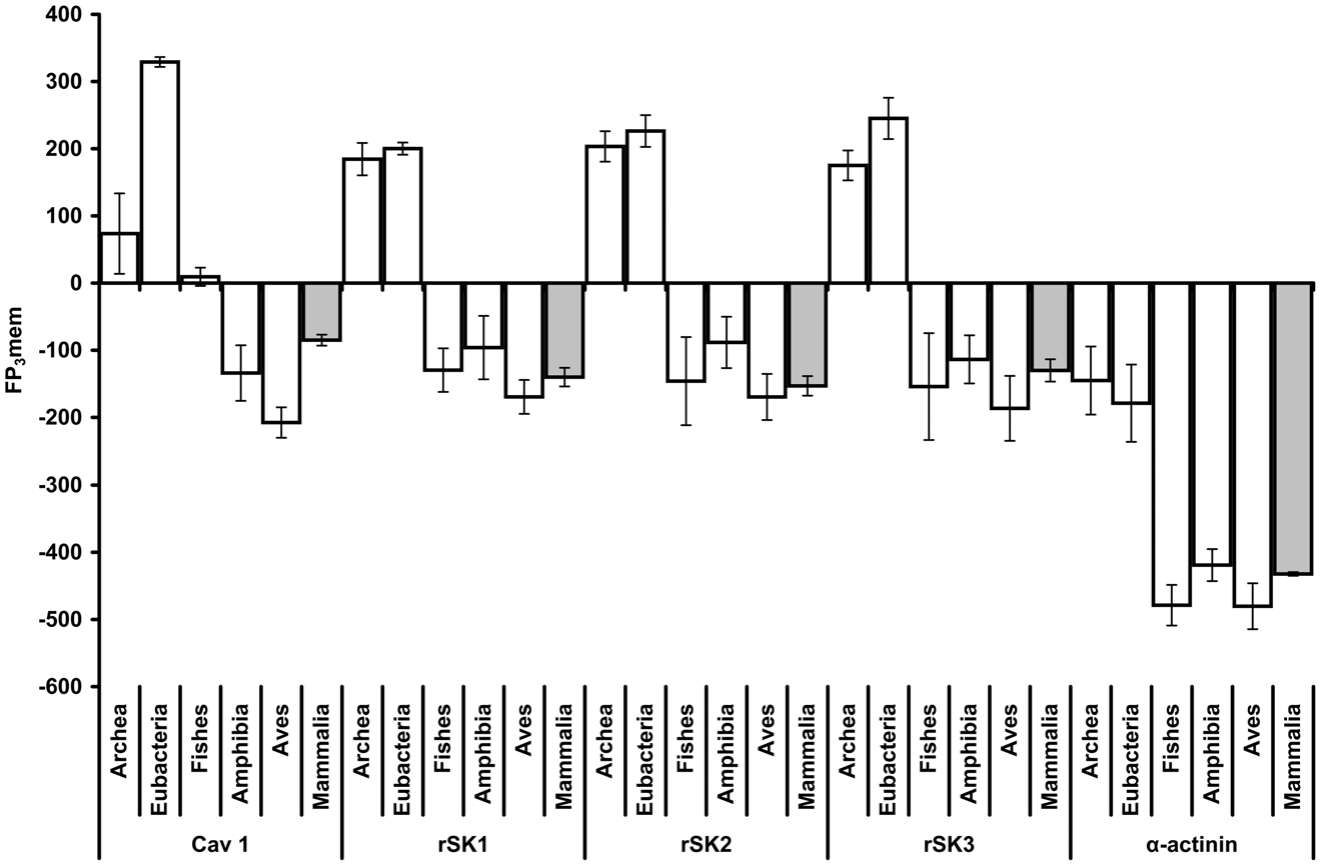
The FP_3_mem values of rSKs, α-actinin and Ca_v_1.2 proteins. Columns indicate the FP_3_mem value for each protein over evolutionary time. The mammalian columns are colored gray for an easier comparison. The error bars designate the SEM.

We use the FP_3_mem score to study the rSKs- α-actinin- Ca_v_1.2 protein system in various organisms (Figure 7). The FP_3_mems of α-actinin homologous proteins are similar to non-membrane proteins in all evolutionary branches. FP_3_mem values are smaller than cut-off and equivalent to zero membrane association in all branches (Figures 5, 6). We consider rSK channel homologous proteins as membrane proteins only in prokaryotes. However, for Ca_v_1.2 channel homologous proteins, in addition to prokaryotes, Fishes and to a less degree Mammalia show higher propensities toward localization in the membrane. This difference in the membrane association of mammalian rSK and Ca_v_1.2 channels may be the reason for the observed fading of SK2 channel presence in the plasma membrane in the absence of Ca_v_ [35] (Figure S2). Because of their high FP_3_mem, we hypothesize that the Ca_v_1.2 channels assist in the membrane localization of SK channels in Mammalia and Fishes.

**Figure 8:**
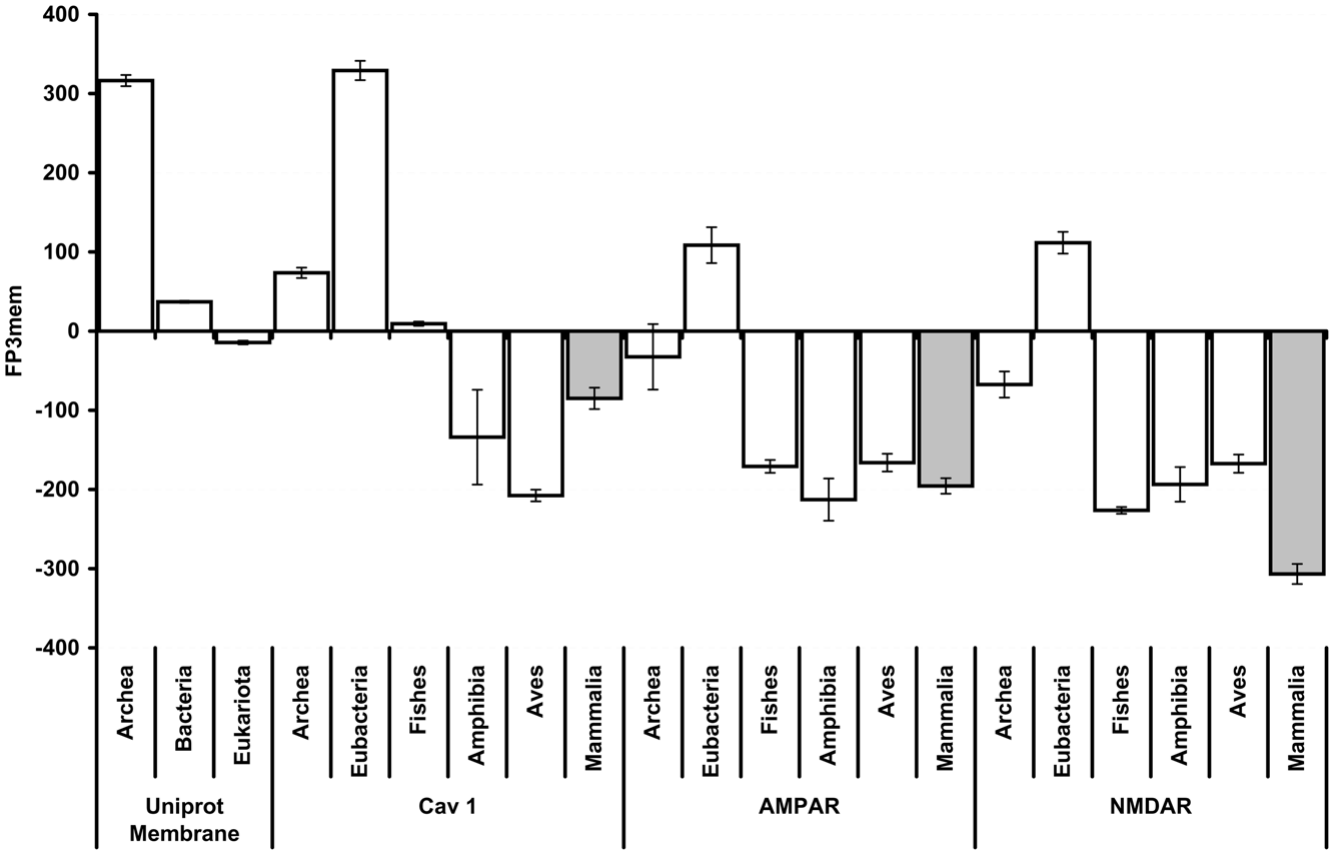
AMPAR and NMDAR co-localize with the Ca_v_ channel. Many membrane proteins co-localize with the Ca_v_ channel. The FP_3_mem values of those proteins are compared with the Ca_v_ FP_3_mem. The FP_3_mem cut-off value for the plasma membrane proteins is set at −31. The average FP_3_mem for the transmembrane proteins of Archea, Bacteria, and Eukaryotes are retrieved from Uniprot and presented for comparison. The error bars indicate the SEM.

Supporting data exist for the presence of Ca_v_1.2 channel in complexes containing AMPAR or NMDAR glutamate receptors [38]. Contrary to rSK channels, none of these receptors depends directly upon the Ca^2+^ influx for activation. Hence, we hypothesize that the reason they accompany the Ca_v_ channel is to localize within the membrane, and that this membrane localization does not occur in the absence of Ca_v_. In order to test this hypothesis, we calculate FP_3_mem for the homologous proteins of these receptors in prokaryotes and vertebrates (Figure 8). We observe that the FP_3_mem of Ca_v_1.2 homologous proteins is higher than the FP_3_mem of the AMPAR and NMDAR homologous proteins, especially in Fishes and Mammalia. This observation supports the proposed hypothesis that these receptors couple the Ca_v_1.2 channel with the aim of localizing within the membrane.

### Conclusion

By creating a score (FP_3_mem) encompassing the biophysical parameters involved in the folding of α-helical transmembrane proteins, we provide a scale for measuring the propensity of protein sequences for localization within the plasma membrane. This parameter distinguishes membrane proteins from non-membrane proteins in various datasets, and powerfully competes with other methods. Furthermore, FP_3_mem quantifies a protein's propensity for becoming an α-helical transmembrane protein. We suggest the difference in this propensity as an underlying reason for the colocalization of SK channels, as well as AMDAR and NMDAR receptors, with the Ca_v_1.2 calcium channel.

## Supporting Information

Figure S1 **The ROC curve.** The sensitivity is plotted against 1-specificity. The bold black filled circle is the cutoff point.
(TIF)

Figure S2 **The histogram of FP3mem value for the SK2 and Cav proteins in Mammalia.** The vertical lines show the FP3mem of mice SK2 and Cav 1.2.
(TIF)

Table S1 **The dimension of studied protein set.**
(DOC)

Table S2 **The PCA extracted coefficients.**
(DOC)

Table S3 **The dimension of tested datasets.**
(DOC)
