# Supplementary figures and images for: A Folding Pathway-Dependent Score to Recognize Membrane Proteins
Source: PLoS One. 2011 Mar 1;6(3):e16778. doi: 10.1371/journal.pone.0016778 (PMC3046963; doi:10.1371/journal.pone.0016778)

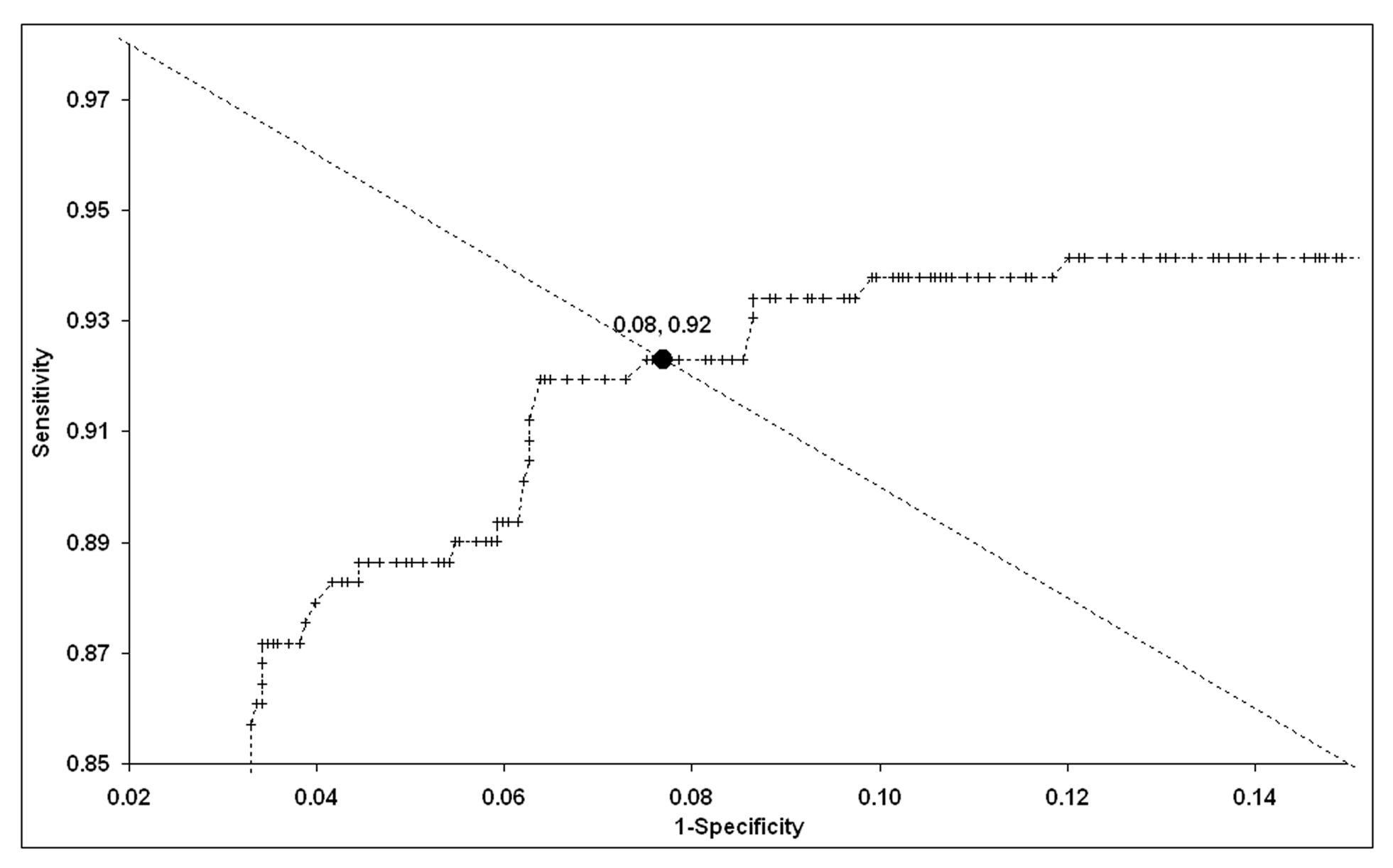

Supplement: Figure S1 — The ROC curve. The sensitivity is plotted against 1-specificity. The bold black filled circle is the cutoff point. (TIF) [file pone.0016778.s001.tif]

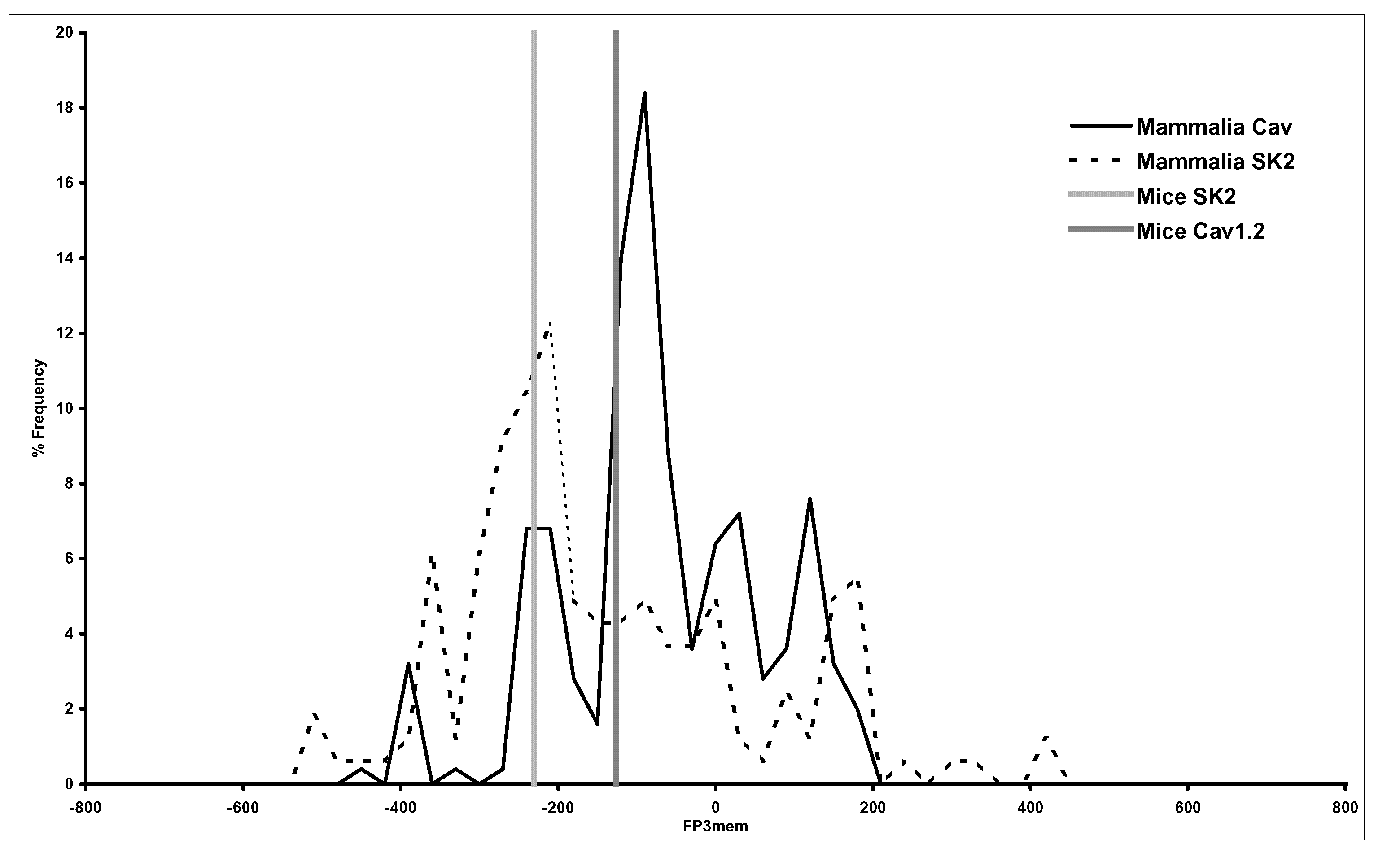

Supplement: Figure S2 — The histogram of FP3mem value for the SK2 and Cav proteins in Mammalia. The vertical lines show the FP3mem of mice SK2 and Cav 1.2. (TIF) [file pone.0016778.s002.tif]
