# Supplementary material for: A Folding Pathway-Dependent Score to Recognize Membrane Proteins
Source: PLoS One. 2011 Mar 1;6(3):e16778. doi: 10.1371/journal.pone.0016778 (PMC3046963; doi:10.1371/journal.pone.0016778)
Supplement: Table S1 — The dimension of studied protein set. (DOC) [file pone.0016778.s003.doc]

**Table S1**. **The dimension of studied protein set.**

|  | **α-actinin** | **Cav 1** | **rSK1** | **rSK2** | **rSK3** | **NMDAR** | **AMPAR** | **Total sequence number** |
| --- | --- | --- | --- | --- | --- | --- | --- | --- |
| **Archea (taxid 2157)** | 10 | 16 | 74 | 51 | 76 | 65 | 40 | 337 |
| **Eubacteria (taxid 2)** | 12 | 100 | 100 | 19 | 18 | 100 | 100 | 460 |
| **Fish (taxid 7898)** | 250 | 250 | 100 | 159 | 126 | 100 | 100 | 1185 |
| **Amphibia (taxid 8292)** | 105 | 56 | 42 | 45 | 46 | 57 | 64 | 515 |
| **Ave (taxid 8782)** | 182 | 47 | 131 | 123 | 120 | 100 | 94 | 897 |
| **Mamalia (taxid 40674)** | 93 | 250 | 174 | 163 | 137 | 100 | 100 | 1117 |
| **Total sequence number** | 652 | 719 | 621 | 560 | 523 | 522 | 498 | 4511 |

The dimension of tested protein set studied in this article based on evolutionary divisions is shown.
