# Supplementary material for: A Folding Pathway-Dependent Score to Recognize Membrane Proteins
Source: PLoS One. 2011 Mar 1;6(3):e16778. doi: 10.1371/journal.pone.0016778 (PMC3046963; doi:10.1371/journal.pone.0016778)
Supplement: Table S2 — The PCA extracted coefficients. (DOC) [file pone.0016778.s004.doc]

**Table S2**. **The PCA extracted coefficients.**

|  | **Component** | | | |
| --- | --- | --- | --- | --- |
|  | **1** | **2** | **3** | **4** |
| **∆Cp** | 0.773 | 0.626 | . | . |
| **∆H(h)** | 0.882 | . | . | . |
| **∆G(h)** | 0.868 | . | . | . |
| **∆Gwif** | . | -0.673 | . | -0.043 |
| **∆G oct** | -0.83 | . | 0.443 | 0.118 |
| **∆∆Gαhw** | . | . | 0.417 | . |
| **∆Cp(h)** | 0.772 | 0.629 | . | -0.075 |
| **kProtw** | 0.685 | . | . | 0.064 |
| **GG4brw** | . | . | . | 0.037 |

These coefficients were used to build up the FP_3_mem.
