# Supplementary material for: A Folding Pathway-Dependent Score to Recognize Membrane Proteins
Source: PLoS One. 2011 Mar 1;6(3):e16778. doi: 10.1371/journal.pone.0016778 (PMC3046963; doi:10.1371/journal.pone.0016778)
Supplement: Table S3 — The dimension of tested datasets. (DOC) [file pone.0016778.s005.doc]

**Table S3. The dimension of tested datasets.**

|  | **TMPDBA** | 231 |
| --- | --- | --- |
| **Steffen Moller** | **A** | 37 |
|  | **B** | 23 |
|  | **C** | 129 |
| **David S. Wishart dataset** | **GLOBUL** | 16623 |
|  | **TMA** | 273 |
| **PDBTM** | **ALFA** | 288 |
|  | **BETA** | 49 |
| **MPtopo** | **1D_helix** | 41 |
|  | **3D_helix** | 131 |
| **eSLDB** | **HUMAN** | 76451 |
|  | **NEMATODE** | 32839 |
|  | **YEAST** | 7367 |
| **PSORTb 3.0** | **Archea** | 763 |
|  | **Bacteria (+)** | 2251 |
|  | **Bacteria (-)** | 7374 |
| **UniProt-Localized in The Membrane** | **Archea** | 1062 |
|  | **Bacteria** | 62326 |
|  | **Eukariota** | 13930 |
|  | **Mamalia** | 8165 |
| **Uniprot-cell fraction** | **Human Soluble** | 1756 |
| **Rohan D Teasdale dataset** | **LOC214PM** | 642 |
|  | **SP376PM** | 226 |
| **Total protein sequence** |  | 232977 |

These datasets were used to evaluate the FP_3_mem score.
